# Supplementary material for: Family functioning but not social capital is associated with better mental health in adolescents affected by violence and displacement by armed conflict in Colombia
Source: Int J Soc Psychiatry. Author manuscript; Available in PMC 2022 Dec 1. (PMC9597145; doi:10.1177/00207640211045417)
Supplement: Supplemental material [file EMS151983-supplement-Supplemental_material.pdf]

Supplementary Material for Tamayo-Aguledo et al paper:

Family functioning but not social capital is associated with better mental health in adolescents affected by violence and displacement by armed conflict in Colombia

NMHS Item	*Original question in Spanish*	*English translation*
m2_p57	¿La mayoría de sus vecinos están dispuestos a ayudar cuando otro vecino tiene una emergencia?	Are most of your neighbours willing to help out when another neighbour has an emergency?
m2_p58	¿Puede confiar en la mayoría de sus vecinos?	Can you trust most of your neighbours?
m2_p59	Si un proyecto de la comunidad no lo beneficia directamente, pero tiene beneficios para muchas otros vecinos, ¿usted contribuiría?	If a community project does not directly benefit you, but benefits many other neighbours, would you contribute?
m2_p60	Si hubiera un problema con el suministro de agua o luz ¿usted y sus vecinos se ayudarían entre si?	If there was a problem with the water or electricity supply, would you and your neighbours help each other?
m2_p61	¿Usted o alguien de su hogar tiene como práctica frecuente participar en alguna actividad en beneficio de la comunidad?	Do you or someone in your household frequently participate in any activity for the benefit of the community?
m2_p61	¿Si a usted se le perdiera la billetera fuera de su casa, cree que se la devolverían?	If you lost your wallet outside your home, do you think it would be returned to you?

Table S1. Summed items used to calculate the cognitive social capital variable

Prompt question:

De las siguientes personas, seleccione aquellas que con las que usted podría discutir sus problemas o le brindarían apoyo si lo necesitara

From the following people, select those with whom you could discuss your problems or provide support if you need it

NMHS Item	*Original item in Spanish*	*English translation*
m2_p55__1	Miembros de su familia	Members of your family
m2_p55__2	Amigos	Friends
m2_p55__3	Vecinos	Neighbours
m2_p55__4	Pareja	Partner
m2_p55__5	Compañero de trabajo o de estudio	Colleague from work or education
m2_p55__6	Otro	Other

Table S2. Summed items used to calculate the individual structural social capital variable

Prompt question:

¿En cuáles de los siguientes grupos participa?

In which of the following groups do you participate?

NMHS Item	*Original item in Spanish*	*English translation*
m2_p56__1	Grupos religiosos	Religious groups
m2_p56__2	Grupos deportivos	Sport groups
m2_p56__3	Grupos políticos	Political groups
m2_p56__4	Grupos culturales	Cultural groups
m2_p56__5	Grupos comunitarios	Community groups
m2_p56__6	Grupos ecológicos	Ecological groups
m2_p56__7	Grupos gremiales	Profession-based groups
m2_p56__8	Grupos étnicos	Ethnic groups
m2_p56__9	Grupos juveniles	Youth groups
m2_p56__10	Grupos de salud	Health groups

Table S3. Summed items used to calculate the group structural social capital variable

	Model 1	*Model 2*	*Model 3*	*Model 4*	*Model 5*
	Unadjusted	+ Demographic confounders	+ Displacement by armed conflict	+ Non-specific violence & harm	+ Remaining social capital / family factors
Structural social capital – support	1.50 (1.06 - 2.12)	1.54 (1.06 - 2.25)	1.48 (1.01 - 2.18)	1.48 (1.01 - 2.18)	1.45 (0.98 - 2.13)
Structural social capital – group membership	1.02 (0.69 - 1.50)	1.02 (0.70 - 1.51)	1.02 (0.69 - 1.51)	1.02 (0.69 - 1.51)	1.06 (0.72 - 1.58)
Cognitive social capital	0.81 (0.69 - 0.97)	0.96 (0.79 - 1.17)	0.95 (0.78 - 1.15)	0.98 (0.85 - 1.14)	0.97 (0.79 - 1.19)
Family functioning	0.90 (0.85 - 0.96)	0.91 (0.86 - 0.97)	0.91 (0.86 - 0.97)	0.91 (0.86 - 0.97)	0.91 (0.85 - 0.97)

Table S4. Results of all analyses repeated to include only those adolescents who reported family ties. Each cell reports the relevant odds ratio and 95% confidence intervals in brackets. The analysis for family functioning and Model 5 are identical as they depend on the presence of a family APGAR score as per the prior analyses but are included for ease of comparison.
